# Ingestional and transgenerational effects of the Fukushima nuclear accident on the pale grass blue butterfly

**DOI:** 10.1093/jrr/rrv068

**Published:** 2015-12-09

**Authors:** Wataru Taira, Atsuki Hiyama, Chiyo Nohara, Ko Sakauchi, Joji M. Otaki

**Affiliations:** The BCPH Unit of Molecular Physiology, Department of Chemistry, Biology and Marine Science, Faculty of Science, University of the Ryukyus, Nishihara, Okinawa 903-0213, Japan

**Keywords:** pale grass blue butterfly, Fukushima nuclear accident, transgenerational effect, internal exposure, ingestional effect, natural selection, radiation resistance, radiation sensitivity, adaptive evolution

## Abstract

One important public concern in Japan is the potential health effects on animals and humans that live in the Tohoku-Kanto districts associated with the ingestion of foods contaminated with artificial radionuclides from the collapsed Fukushima Dai-ichi Nuclear Power Plant. Additionally, transgenerational or heritable effects of radiation exposure are also important public concerns because these effects could cause long-term changes in animal and human populations. Here, we concisely review our findings and implications related to the ingestional and transgenerational effects of radiation exposure on the pale grass blue butterfly, *Zizeeria maha*, which coexists with humans. The butterfly larval ingestion of contaminated leaves found in areas of human habitation, even at low doses, resulted in morphological abnormalities and death for some individuals, whereas other individuals were not affected, at least morphologically. This variable sensitivity serves as a basis for the adaptive evolution of radiation resistance. The distribution of abnormality and mortality rates from low to high doses fits well with a Weibull function model or a power function model. The offspring generated by morphologically normal individuals that consumed contaminated leaves exhibited high mortality rates when fed contaminated leaves; importantly, low mortality rates were restored when they were fed non-contaminated leaves. Our field monitoring over 3 years (2011–2013) indicated that abnormality and mortality rates peaked primarily in the fall of 2011 and decreased afterwards to normal levels. These findings indicate high impacts of early exposure and transgenerationally accumulated radiation effects over a specific period; however, the population regained normality relatively quickly after ∼15 generations within 3 years.

## INTRODUCTION

The Fukushima Nuclear Power Plant (NPP) accident is the second largest nuclear accident next to the Chernobyl accident in the history of mankind. Epidemiological and physiological studies on humans following the Chernobyl accident that assessed infant leukemia [1–6], infant mortality [7, 8], blood cell counts [9], and thyroid cancer [10, 11], and ecological studies on wild organisms, including plants, insects, birds and small mammals [12–21], have accumulated evidence that indicates a number of biological impacts of the accident. In these studies, ingestional risks and transgenerational effects are discussed. Although this body of research strongly suggests a causal relationship between the Chernobyl accident and the observed biological changes, experimental studies combined with field work that clearly demonstrate a causal involvement of the accident are scarce.

The biological impacts of the Fukushima accident have been demonstrated in several studies. Census studies indicate that the number of birds and insects, especially butterflies, has decreased in contaminated areas [22–24]. For example, the reproductive decline of two species of birds, barn swallow [25] and goshawk [26], has been reported. Morphological abnormalities have been identified in aphids in contaminated areas [27]. Effects on other organisms have also been reported: for example, low numbers of blood cells have been reported in Japanese monkeys living in Fukushima [28]. In addition, experiments have determined that the leaf development of the rice plant has been affected by radiation exposure in Iitate Village, one of the highly contaminated localities, and that this has been accompanied by changes in the expression patterns of DNA repair genes [29].

**Fig. 1.**
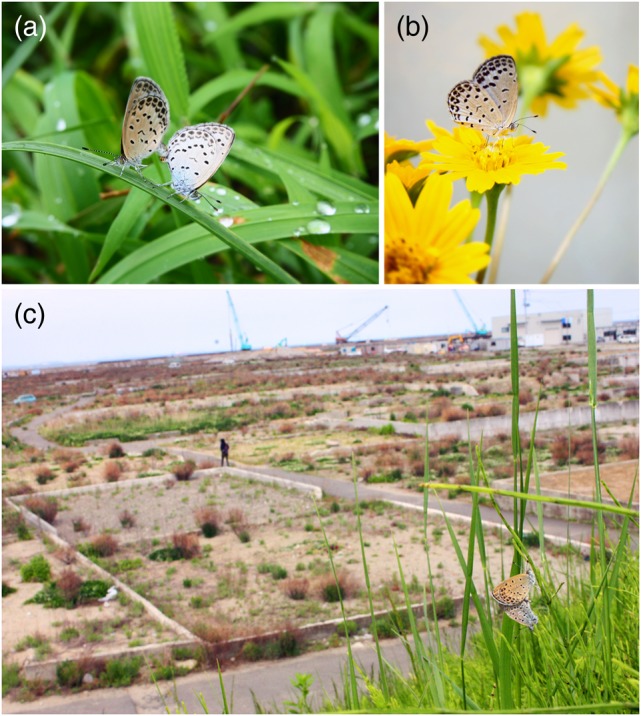
Pale grass blue butterfly in the Tohoku district. (**a**) A pair of pale grass blue butterflies in Minami-soma City, Fukushima Prefecture. Pictured in August 2014. (**b**) An adult individual caught in Okuma Town, Fukushima Prefecture and pictured in the laboratory, Okinawa Prefecture. Pictured in August 2014. (**c**) A pair of pale grass blue butterflies in Yuriage, Natori City, Miyagi Prefecture. Pictured in May 2013.

In the previous studies, we have used the pale grass blue butterfly as a model organism for physiology, genetics, developmental biology and evolutionary biology [30–33] and as an environmental indicator for radioactive contamination caused by the Fukushima nuclear accident [34–40] (Fig. 1). The use of lepidopteran and other insects in radiation research is not new. A number of lepidopteran insects are major agricultural pests; thus, many studies have focused on how to exterminate these insects [41, 42]. The larvae of the pale grass blue butterfly feed on the weed, *Oxalis corniculata*, which is distributed worldwide and is difficult to eradicate from farmland. Thus, the pale grass blue butterfly is a beneficial insect in agriculture. It is the most prosperous butterfly in Japan, both in rural and urban areas, including Tokyo [43–46].

Our butterfly research on Fukushima initiated discussions regarding the biological impacts of the NPP accident [34–40, 47–49]. In a meeting of the Kyoto University Research Reactor Institute in Kumatori, Japan, which was held on 10–11 August 2014, we presented novel data regarding the ingestional and transgenerational effects of the Fukushima nuclear accident on the pale grass blue butterfly. Internal exposure primarily occurs through the ingestion of contaminated foods and is most likely one of the greatest public concerns in Japan, especially for individuals who live in contaminated areas close to the NPP. Evaluations of ingestional effects are especially important for allowing the public to make informed decisions regarding what to eat, where to live, and how to live in general. Over the long term, the transgenerational or heritable effects of ingesting contaminated foods constitute a major public concern because one generation's decisions regarding what to eat may affect their offspring's generation. Knowledge of ingestional and transgenerational effects is also important for developing reasonable predictions of the future consequences for animal and human populations.

In this meeting report, we first discuss certain basic but easily overlooked considerations and then illustrate the major points of our results regarding ingestional and transgenerational effects. We also present interpretations of the morphological examinations and mathematical models that describe the biological responses (i.e. abnormalities and deaths) to the amount of cesium radioactivity ingested. We subsequently address representative questions that have been received in this and other meetings after presenting our results. A concise summary and discussion through questions and answers will help readers understand what is known and unknown and the implications of our findings from butterfly studies.

## BASIC CONSIDERATIONS REGARDING RADIATION-INDUCED DAMAGE

### Internal exposure through ions and microparticles

Although a number of different radionuclides were released from the Fukushima Dai-ichi NPP into the environment [50, 51], there are two distinct types of radioactive materials that potentially cause internal exposure. One type is water-soluble ions of radionuclide species that emit ionizing radiation, such as cesium and iodine ions [50], and the other type is water-insoluble microparticles containing cesium, uranium and other radionuclide species; both types were released in the early stages of the accident [52–54]. These two types of released materials are expected to cause repeated exposures inside the body, and the effects of β-ray doses are most likely significant [55] in both cases. However, they behave differently within the body after ingestion.

Although the ways in which artificial microparticles influence biological systems are entirely unknown, they likely have provided a significant contribution to the biological impacts of the accident. Ingestion of the metallic microparticles that were heavily deposited on the surface of host plant leaves, regardless of the radioactivity of these particles, may also contribute to the toxicity of the host plant leaves collected from contaminated areas in our internal exposure experiments [34, 35]. Metallic particles that are ingested along with other organic materials may cause stress and immune responses in butterfly larvae and other animals living in contaminated areas. The toxicity of metallic microparticles from the NPP explosion has rarely been discussed in the scientific literature. The possibility of stress and immune responses induced by metallic microparticles implies that investigation of the biological impacts of the Fukushima nuclear accident cannot be confined to studies of radiation biology. Moreover, radioactivity and metallic microparticles may produce a synergistic adverse effect on organisms.

### Radiation-induced damage and heritability

First, the meaning of ‘radiation-induced damage’ must be clarified to avoid miscommunication. Radiation-induced damage on biological systems varies; however, it may be classified into one of the following two categories for convenience: germline damage (heritable damage) and somatic damage (non-heritable damage).

Somatic damage represents damage to somatic cells, and it is not heritable. Somatic damage may generally be referred to as physiological damage. Although damaged genetic material (i.e. DNA) in somatic cells may be referred to as ‘genetic’ (or ‘epigenetic’) damage at the cellular level because somatic cell genetic material that is damaged by environmental stressors is replicated and passed down to somatic cells following somatic cell division, this damage is not heritable at the individual level, and thus, in this sense, it remains physiological. In our experimental systems, ingestional effects were classified as physiological damage to the pale grass blue butterfly; however, we have not yet evaluated the significance of ingestional genetic damage.

As distinct from somatic damage, ‘germline damage’ typically refers to the genetic damage of germline cells. Because damage often occurs to the DNA of germline cells, the damage is heritable by the next and subsequent generations. In classical ‘forward’ genetics, radiation-induced or chemically induced mutagenesis is employed, and mutants are generated via the random introduction of DNA damage. We utilized this method to create mutants of the pale grass blue butterflies using the DNA-damaging agent ethyl methane sulfonate (EMS) [31]. Our results suggest that the radioactive materials released immediately after the explosions at the NPP caused both somatic damage and DNA damage to the germline cells of the butterflies that was similar to EMS-induced damage. We may be able to demonstrate these types of changes at the DNA level in future genomic analyses.

### Epigenetic changes and genomic instability

Ionizing radiation is believed to cause major DNA damage (i.e. DNA strand breaks, base-pair changes and other types of damage that results in base-pair changes). However, DNA may be modified without base-pair changes, such as by methylation, which could alter gene expression patterns. Furthermore, histone modifications may alter gene expression patterns without changing DNA sequences. When a change is introduced to the germline cells by environmental stressors and is observed to be transgenerational or heritable, the change is referred to as ‘epigenetic’. Exposure to radiation could cause these changes, although this has not yet been rigorously demonstrated. The number of generations through which transgenerational damage is transferred may depend on the nature of the biochemical changes associated with the damage. Adding further complexity, genomic instability may occur in subsequent generations [56–59]. Epigenetic changes and genomic instability may have occurred in the pale grass blue butterfly; however, we have not yet obtained evidence for these effects.

### Non-DNA damage and heritability

Non-genetic materials (i.e. RNA, proteins and other materials) in somatic cells may also be damaged, and this phenomenon is rarely discussed in radiation biology. Although this damage could greatly affect the homeostasis of an individual, it is not heritable. However, parental olfactory experiences have recently been demonstrated to be transgenerational [60]. This surprising discovery implies that somatic damage by radiation exposure might also be transgenerational. We discovered that the small forewing size induced by internal exposure is likely passed down to the next generation, although there may not be associated germline damage [38].

Furthermore, non-genetic material (i.e. mainly proteins and RNA) in germline cells may also be damaged. Damage of non-genetic materials is not directly heritable; however, it could affect the quality of eggs and sperms and may affect the offspring generation. Thus, the damage of non-genetic materials may be transgenerational.

### DNA damage in normal development

Certain types of DNA damage, such as DNA digestion or fragmentation, may be endogenously introduced over the course of apoptosis, denucleation, genetic recombination, and other processes of terminal cellular differentiation. These types of DNA damage are programmed as normal processes during development and should be distinguished from radiation-induced DNA damage through appropriate control experiments and by understanding normal development.

### DNA damage and aging

Although the aging process in mammals may not be readily applicable to insects, it has recently been demonstrated that accumulated DNA damage caused by reactive oxygen species in hematopoietic stem cells greatly contributes to aging in mammals [61–63]. Reactive oxygen species are spontaneously produced by ionizing radiation. Aging-related damage is physiological damage, and this damage is passed down to daughter cells from stem cells inside the body. Because reactive oxygen species that occur spontaneously contribute to aging, and the aging process is linked to chronic disease processes [64–68], it is reasonable to speculate that ionizing radiation, even at low doses, contributes to the advancement of aging and may promote chronic diseases in general in mammals and other animals.

### Molecular damage and macroscopic phenotypes

Radiation-induced damage is an event that occurs at the molecular level, and it does not necessarily result in phenotypic changes or physiological effects at the individual level. Organisms or cells damaged at the molecular level may not exhibit abnormalities at the individual level. In that case, these molecular changes are not significant at the level of physiological function. Having said that, phenotypic changes detected at the individual level are a clear indication that molecular changes have occurred, and these health effects (i.e., phenotypic changes) are considered the most important of these biological changes.

Thus, molecular detection should accompany the detection of phenotypic or physiological changes at the individual (organismal) level to be functionally significant, and they can eventually be explained by the molecular changes of the system. A mechanistic explanation will help researchers and the general public to more deeply understand the nature of radiation exposure.

## BASIC CONSIDERATIONS REGARDING THE NATURAL HISTORY OF THE BUTTERFLY

### Life cycle and overwintering

The pale grass blue butterfly completes one life cycle in ∼1 month under our standard rearing conditions. In subtropical regions, such as Okinawa, adults can be found throughout the year, although they are not frequently observed during the winter season. How *Z. maha* overwinters in the Fukushima area may help us to understand the manner in which *Z. maha* was irradiated immediately after the explosions at the Fukushima Dai-ichi NPP.

The last instar for summer larvae is the fourth instar. The pale grass blue butterfly overwinters as larvae. The overwintering larvae have been suggested to be primarily fourth instar, and they become fifth instar in the spring [43, 44]. However, a more detailed observation has indicated that the fourth instar larvae in the winter season die because of a lack of fresh leaves, whereas the second and third instar larvae survive by eating emerging buds and growing slowly during the winter season [45]. At the beginning of spring, these larvae become fourth instar larvae and subsequently fifth instar larvae [45]. This overwintering strategy is consistent with the fact that the spring morph is larger than the summer morph.

If this observation is correct, then at the time of the Fukushima Dai-ichi NPP collapse, *Z. maha* was most likely in the third instar stage. These larvae certainly ate many leaves immediately after the power plant explosions as they progressed to the fourth instar and subsequently the fifth instar. The amount of contaminated leaves ingested by the first generation was most likely as large as or slightly larger than the amount of contaminated leaves ingested by subsequent generations. The contamination level was also highest and contained many short-lived radionuclides. Therefore, it is clear that the first generation of this butterfly after the accident was highly exposed and most likely the most irradiated among all generations, both externally and internally.

**Fig. 2.**
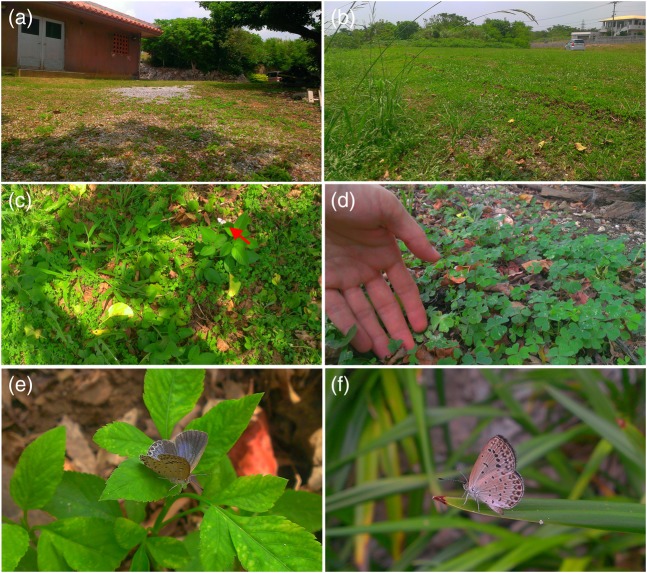
Pale grass blue butterfly and its host plant in Okinawa (Nanjo City) in August 2015. (**a, b**) A typical habitat, where numerous butterflies are found: the ground is stony and covered with the host plant. (**c**) A male butterfly flying close to the ground of the host plant field (red arrow). The butterfly is flying at the height of the shoes of the photographer. The shadow of the photographer (who is standing straight) can be observed. (**d**) The host plant with a hand of the photographer, indicating the size of the plant. (**e, f**) A female butterfly in this habitat.

Because many of the radionuclides from the NPP had short half-lives of less than a few days, they may not have been significantly ingested. However, they likely contributed to external exposure, although an important exception is ^131^I, which has an eight-day half-life. Therefore, external exposure may have been more severe than internal exposure for the first-generation butterflies. Because this butterfly species lives close to the ground throughout its lifecycle (Fig. 2), external exposure to β rays and γ rays most likely contributed to its biological damage [55].

### Butterfly and host plant association

The pale grass blue butterfly is monophagous, and it eats the creeping wood sorrel (*Oxalis corniculata* L. (family: Oxalidaceae); *Katabami* in Japanese), which is an indigenous plant in Japan [69, 70]. In Japanese, variations in the phenotype of the *Katabami* plant are often referred to by names such as *Tachi-katabami*, *Usuaka-katabami*, *Aka-katabami* and *Ke-katabami*; however, these are all variants of *O. corniculata*. In our experiments, including internal exposure experiments that used host plant leaves from contaminated areas, *O. corniculata* (*i.e.* *Katabami* and, much less frequently, *Tachi-katabami*) was always used. We cannot completely exclude the possibility that *Usuaka-katabami* may have been accidentally included, although its inclusion is likely to have been rare.

Other *Oxalis* species from naturalized immigrants are observed in Japan [69, 70]: *O. articulata* (synonym: *O. rubra*; *Imo-katabami*), originating from South America; *O. bowiei* (Bowie's woodsorrel; *Hana-katabami*), originating from southern Africa; *O. corymbosa* (synonym: *O. debilis*; violet woodsorrel; *Murasaki-katabami*), originating from South America; and *O. stricta* (synonym: *O. dillenii*; European woodsorrel; *Ottachi-katabami*), originating from North America. In addition, *O. fontana* (yellow woodsorrel; *Ezo-tachi-katabami*) is considered to be a synonym of *O. stricta*. These species do not serve as host plants for *Z. maha* in the field, with the potential exception of *O. stricta* [43, 44]. We suggest that the pale grass blue butterfly does not lay eggs on this plant in the field, although we have not yet rigorously examined this possibility. In our experiments, including the internal exposure experiments that used host plant leaves from the contaminated areas, we always excluded these non-host *Oxalis* species.

This strict monophagous nature of *Z. maha* is remarkable, and as long as *Katabami* was provided, the larvae grew normally in our experiments, regardless of the collection site, unless it was contaminated. Furthermore, as an added precaution, we avoided non-regular forms of *Katabami*, although they also belong to *O. corniculata*. We believe that *Katabami* (the regular form of *O. corniculata*) from anywhere in Japan is fundamentally indistinguishable for *Z. maha* and serves as the natural host plant of this butterfly.

It is again important to recognize that the host plant of this butterfly species is generally <10 cm in height above the ground (Fig. 2), which indicates that the larvae and pupae were exposed to β rays in addition to γ rays from the ground surface. Indeed, larvae often overwinter on the ground surface associated with the host plant, which increases the likelihood of β-ray exposure for the larvae [55].

## MAJOR FINDINGS OF THE PAPERS

### Hiyama *et al.* (2012) *Scientific Reports* [34]

**Fig. 3.**
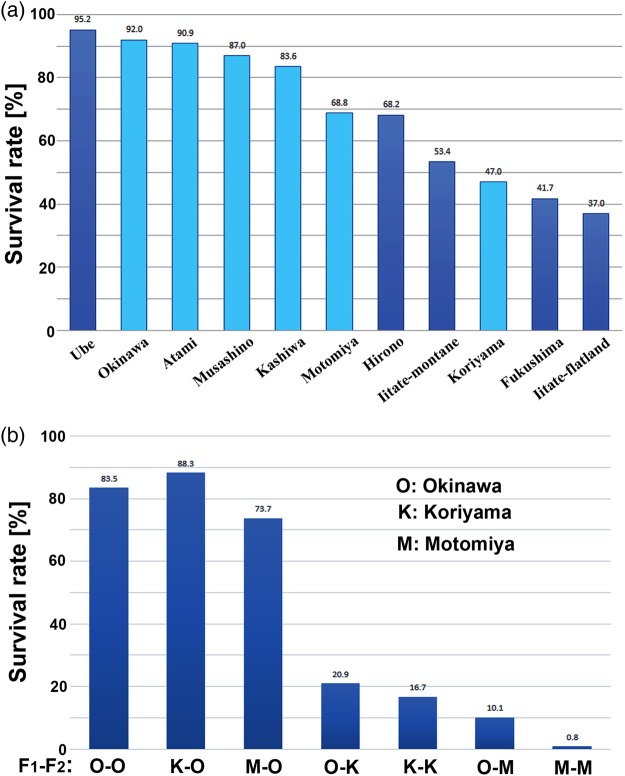
Survival rate (percentage survival) of the internal exposure experiments. (**a**) The effects on the first generation, which include relatively high contamination levels [34] (the first set of experiments, shown in dark blue bars) and relatively low contamination levels [38] (the second set of experiments, shown in light blue bars). (**b**) The effects on the second generation [38]. For example, ‘O-K’ indicates that leaves from Okinawa were given in the first generation (defined as F_1_) and leaves from Koriyama were given in the second generation (defined as F_2_).

This paper was our first report on the Fukushima NPP accident. The internal exposure experiment was performed using contaminated leaves collected from Hirono Town, Fukushima City, Iitate Village (flatland) and Iitate Village (montane region). Leaves from Ube City (Yamaguchi Prefecture) that were not contaminated were also collected and used for comparison. Our results indicated a dose-dependent (locality-dependent) response of mortality (and abnormalities) (Fig. 3a). The morphological abnormalities identified in this experiment were similar to those of the adult samples collected from the contaminated areas. In a different set of experiments, we obtained two generations of offspring (defined as the F_1_ and F_2_ generations) from the field-caught females, and we demonstrated that certain abnormal traits identified in the F_1_ generation were inherited by the F_2_ generation. We further observed that the forewing size was reduced in the internally irradiated individuals. Note that the forewing size is used as an indicator of body size in lepidopterology. A smaller forewing size was also identified in the field-caught individuals collected in the spring (May) of 2011. Thus, we demonstrated reductions in forewing size as a physiological (somatic) stress response to irradiation.

### Hiyama *et al.* (2013) *BMC Evolutionary Biology* [35]

This correspondence paper was published to answer frequent questions that we have received from around the world. We discussed several potential interpretations of the results from the internal exposure experiment and concluded that the pollutants released from the collapsed nuclear reactors were the most likely cause of the observed death and abnormalities. The spatio-temporal gradients of the abnormalities and deaths identified in this paper supported such an interpretation of the impacts of the NPP accident on this butterfly.

### Iwata *et al.* (2013) *Scientific Reports* [31]

This paper reports the results of a mutagenesis experiment in which we produced novel wing color pattern phenotypes in the pale grass blue butterfly using EMS. It is important to note that EMS selectively and effectively introduces single base-pair changes at random in DNA. In the mutagenesis protocol, EMS was fed only to the larvae of the P (F_0_) generation and induced damage in both the somatic and germline DNA of these larvae. From the damaged germline cells, the offspring generation was produced, and their bodies were constructed by mutant somatic cells. The mutant phenotypes obtained were similar to the phenotypes displayed by individuals and the offspring generation in the contaminated areas, which suggests that the Fukushima individuals may have experienced DNA damage.

### Nohara *et al.* (2014) *Scientific Reports* [35]

The consequences of ingesting radioactive materials were further investigated by experimentally determining the amount of leaves that an average larva consumes, calculating the amount of radioactive materials ingested by a larva and applying mathematical models to these data. The dose–response curve obeys a power function and exhibits a sharp increase in response for a small increase in dose. According to the power function equation, the calculated half mortality dose (the dose required to cause death in 50% of the individuals in the treated group; fundamentally equivalent to the median lethal dose, LD_50_) was 1.9 Bq/body (1900 mBq/body), whereas the calculated half abnormality dose (the dose required to cause death or morphological abnormality in 50% of the individuals in the treated group, which is fundamentally equivalent to the median toxic dose, TD_50_) was 0.76 Bq/body (760 mBq/body). We measured cesium activity directly from pupae and demonstrated that the accumulation and retention values of ingested radioactivity were higher at low-dose levels of ingestion compared with at high-dose levels.

### Taira *et al.* (2014) *Journal of Heredity* [37]

This review paper (more precisely, a symposium paper) presents newly performed and detailed analyses of some previously described data. A genetic cross experiment concretely demonstrated in this paper indicated that an abnormal wing color pattern trait was inherited in a Mendelian fashion.

This paper also discusses the results of correlation analyses between the quantitative abnormal traits (i.e. forewing size, abnormality and mortality rates, and eclosion time) in the P and F_1_ generations and the factors associated with the collection locality (i.e. ground radiation dose and distance from the NPP) in the spring (May) and fall (September) of 2011 (see Table 1 in Taira *et al.* (2014) [37]). These findings are discussed below in the section Interpretations of Morphological Examinations.

**Table 1.** Mathematical models with AIC to fit all available data points

Model	Function	AIC
Linear model	f(x)=3.2x+30	104.78
Logistic model (3 parameters)	$f(x)=\frac{73}{1+e^{-3.9(x-0.27)}}$	94.01
Logistic model (4 parameters)	$f(x)=-1018+\frac{1085}{1+e^{-14(x+0.21)}}$	91.60
Log-logistic model (3 parameters)	$f(x)=\frac{85}{1+e^{-0.43(log(x)-log0.11)}}$	84.90
Log-logistic model (4 parameters)	$f(x)=3.4+\frac{78}{1+e^{-0.50(log(x)-log0.11)}}$	86.76
Gompertz model (3 parameters)	$f(x)=73e^{-e^{-3.0(x-0.11)}}$	93.59
Gompertz model (4 parameters)	$f(x)=37+73e^{-e^{1.5(x+85)}}$	113.68
Weibull model (type 1; 3 parameters)	$f(x)=108e^{-e^{-0.21(log(x)-log0.079)}}$	85.41
Weibull model (type 1; 4 parameters)	$f(x)=7.1+80e^{-e^{-0.33(log(x)-log0.054)}}$	86.49
Weibull model (type 2; 3 parameters)	$f(x)=76(1-e^{-e^{-0.35(log(x)-log0.22)}})$	84.76
Weibull model (type 2; 4 parameters)	$f(x)=0.44+76(1-e^{-e^{-0.35(log(x)-log0.22)}})$	86.76
Power function model	$f(x)=53x^{0.17}$	88.13

Furthermore, we propose Postulates of Pollutant-induced Biological Impacts in reference to Koch's Postulates of Infectious Disease. The postulates include six requirements to prove pollutant-induced biological impacts: (i) spatial relationship, (ii) temporal relationship, (iii) direct exposure, (iv) phenotypic variability or spectrum, (v) experimental reproduction (external exposure) and (vi) experimental reproduction (internal exposure). Our *Z. maha* system meets all six requirements, whereas no other system appears to fulfill these requirements to date. Therefore, based on the postulates, we conclude that pollutant-induced biological impacts have rigorously been demonstrated in *Z. maha*.

### Nohara *et al.* (2014) *BMC Evolutionary Biology* [38]

In the internal exposure experiment performed in Hiyama *et al.* (2012) [34] (and its detailed analysis in Nohara *et al.* (2014) [35]), we used leaves contaminated at relatively high levels, whereas in the current paper, we used leaves contaminated at relatively low levels. We collected leaves from the Tokai district (Atami City), the Kanto district (Musashino City and Kashiwa City) and the Tohoku district (Koriyama City and Motomiya City), where people live as if the nuclear accident had not occurred. We discovered that, even at low doses, some butterfly individuals died (Fig. 3a). The data from these five low-level localities [38] and that from the six localities [34] can be coherently understood mathematically, as is discussed in the Mathematical Models section.

We also examined the effects on the second generation. In a different study, we successfully performed artificial breeding experiments in up to at least the 10th generation [32]; thus, the procedures for genetic crosses have been established in this species. However, to produce the second generation, morphologically healthy adults were used, meaning that these normal adults were selected for radiation resistance.

We determined that the second generation was more sensitive to contamination; however, this finding was mostly (but not entirely) independent of ingestion of contaminated or non-contaminated leaves in the first generation (Fig. 3b). For example, even in the Okinawa group, the survival rate decreased from 92.0% in the F_1_ generation to 83.5% in the F_2_ generation. In general, the laboratory-reared generation is more prone to stress (because of a lack of environmental stress and its associated natural selection); thus, these data are not surprising. A comparison between the Okinawa(F_1_)–Motomiya(F_2_) group and the Motomiya(F_1_)–Motomiya(F_2_) group indicated that the effect from the F_1_ generation was detectable but modest. However, a reverse comparison, such as between the Koriyama(F_1_)–Okinawa(F_2_) group and the Okinawa(F_1_)–Koriyama(F_2_) group, demonstrated a large difference, which indicates that the F_1_–F_2_ order is important. Thus, the contamination level in the second generation has a greater influence on the survival of larvae.

Importantly, most of the second-generation larvae that consumed contaminated leaves died; however, when the larvae consumed non-contaminated leaves, most of them did not die and eclosed to morphologically normal adults (Fig. 3b). We admit that these surviving individuals in the second generation (as well as in the first generation) may not be physiologically healthy, despite their normal morphology. Nevertheless, such all-or-none–like responses to contaminated leaves underscore the importance of ingestional effects on animal health.

However, these results do not indicate that eating non-contaminated food is sufficient to circumvent the effects of radiation stress, because in this experiment, the external exposure was minimized and the genetic effect was virtually ignored. Even in our rearing conditions, the air in the container may have been contaminated by radioactive materials from the leaves, and larvae on the leaves may have been externally exposed to radiation from the contaminated leaves; however, these exposures were unavoidable.

The effect of ingesting contaminated leaves in the first generation was manifested in a small forewing size in the first and second generations. Based on this correlation analysis, the effect of radiation stress was likely transgenerational; however, the effects were not clear in the forewing size comparisons of the Koriyama(F_1_)–Okinawa(F_2_) group (or the Motomiya(F_1_)–Okinawa(F_2_) group) with the Okinawa(F_1_)–Okinawa(F_2_) group. We do not know how this body-size effect occurs; however, this effect may be an indication of growth retardation.

An increased sensitivity in females to ingested doses was detected, and this may have resulted from the inherently larger size of the females. A large size requires greater amounts of ingestion over a relatively longer period, which leads to longer radiation exposure. Additionally, females may require more food for the egg production process.

### Hiyama *et al.* (2015) *BMC Evolutionary Biology* [39]

**Fig. 4.**
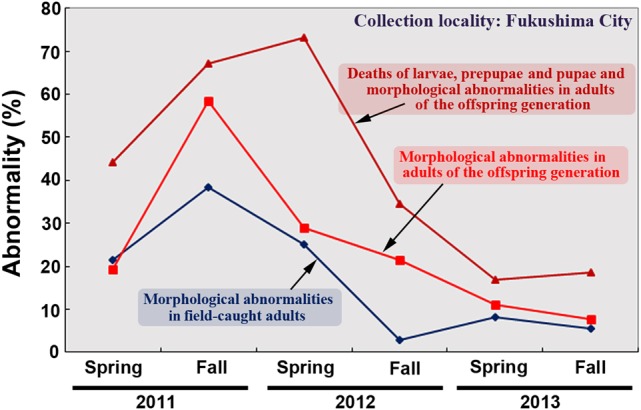
Time course of the three types of ‘abnormality rates’ (percentage abnormal) of butterflies from Fukushima City over 3 years (2011–2013). Peaks in abnormality rates are present in the fall of 2011 and the spring of 2012. All of the abnormality rates then decrease to a normal level. The normal abnormality rates of adults are ∼10% or less [36, 39]. In Hiyama *et al.* (2015) [39], ‘morphological abnormalities of field-caught adults’ is defined as the ‘adult abnormality rate of the P generation’ or ‘aAR(P)’. ‘Morphological abnormalities of adults in the offspring generation’ is defined as the ‘adult abnormality rate of the F_1_ generation’ or ‘aAR(F_1_)’. ‘Deaths of larvae, prepupae and pupae and morphological abnormalities of adults in the offspring generation’ is defined as the ‘total abnormality rate’ or ‘tAR(F_1_)’.

This paper contains detailed accounts of the 3-year dynamics of abnormality and mortality rates of the pale grass blue butterfly in the contaminated areas. The data were analyzed from various aspects, but the conclusion is simple. The abnormality rates in adults peaked in the fall (September) of 2011 or the spring (May) of 2012 and subsequently decreased to normal levels (Fig. 4). This dynamic can be explained by transgenerational accumulation of damage that was then eliminated by natural selection. Because damage occurs by chance within individual organisms (for example, a larva behind a rock may not be exposed at a high level of radiation immediately after the accident), damaged individuals might have been negatively selected, and non-damaged individuals might have survived. Additionally, among the damaged organisms, certain individuals may have been stronger than others, which could have resulted in adaptive evolution. More than 15 generations appear to be necessary for offspring to become resistant to radiation. Many pale grass blue butterflies are currently found in the Tohoku district, and they are morphologically normal (Fig. 1).

Interestingly, a peak of ‘morphological abnormality in field-caught adults’ and a peak of ‘morphological abnormalities in adults of the offspring generation’ both occurred in the fall (September) of 2011, whereas a peak of ‘deaths of larvae, prepupae and pupae and morphological abnormalities in adults of the offspring generation’ occurred in the spring (May) of 2012 (Fig. 4). This peak lag of the latter appears biologically reasonable. Natural selection would work on all developmental stages; however, it is expected that it would produce normal adults as quickly as possible.

### Taira *et al.* (2015) *Scientific Reports* [40]

This paper re-examined the previous finding that the Fukushima butterflies were smaller in size (i.e. forewing size as a representative of body size) than those of the southern populations (in Ibaraki Prefecture and Tokyo) and also those of the northern population (in Miyagi Prefecture) [34], immediately after the accident. Data from 2012 and 2013 revealed that Fukushima butterflies did not show statistically significant differences from those of most of the other populations throughout Japan. Interestingly, the forewing size of the Fukushima population was significantly larger than that of the Aomori and Miyagi populations (i.e. northern populations). Furthermore, the size gap was identified as occurring within the Miyagi Prefecture but not in the Fukushima Prefecture. Thus, this paper strongly supported the idea that the previous forewing size reduction was caused by radiation stress from the Fukushima nuclear accident.

## INTERPRETATIONS OF MORPHOLOGICAL EXAMINATIONS

**Fig. 5.**
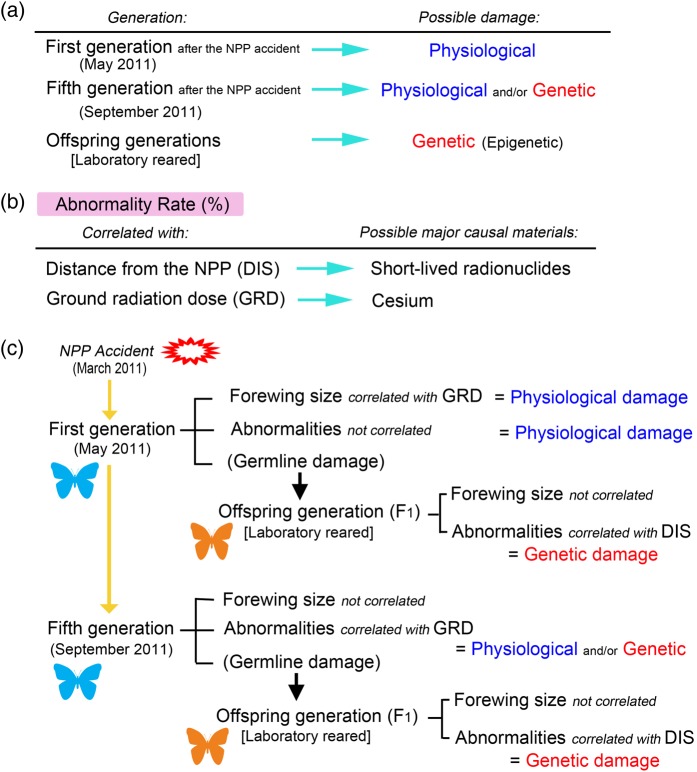
Interpretations of morphological examinations. (**a**) Judgment criteria regarding the nature of damage based on the generations examined. All of the morphological changes in the first generation can be attributed to physiological damage because the first generation directly received irradiation. Morphological changes observed in the laboratory reared (F_1_) generations from the first and fifth generations can be attributed to genetic damage because physiological damage did not occur in the clean laboratory environment; however, some of these changes may have been epigenetic. Morphological changes in the fifth generation may be a mixture of physiological and genetic damages. (**b**) Judgment criteria regarding the potential major causal materials. When abnormality rates are correlated with distance from the NPP, these abnormalities can be attributed to short-lived radionuclides because they are distributed in a concentric fashion. When abnormality rates are correlated with the measured ground radiation doses, these abnormalities can be attributed to the radioactive cesium species. (**c**) Summary of morphological examinations. Physiological (somatic) damage in the first generation is clearly detected. Genetic (germline) damage in the first and fifth generations is also clear, based on the results of the offspring generations. Genetic damage is likely caused by short-lived radionuclides, based on the correlation with distance. Abnormalities in the first generation may be caused by both short-lived radionuclides and cesium, which did not exhibit a clear correlation. Abnormalities in the fifth generation may primarily be caused by cesium.

We examined the morphology of many adult individuals from a number of generations; however, our basic findings are illustrated here by focusing on the first and fifth generations (Fig. 5). Interpretations of the morphological examinations are based on the following two sets of judgment criteria.

The first criterion is that physiological or genetic damage can be judged based on generations (Fig. 5a). All of the morphological changes in the first generation after the NPP accident can be attributed to physiological damage, because these organisms were directly irradiated. Similarly, our internal exposure experiment examines physiological damage. However, all of the morphological changes in the offspring generations reared in the laboratory can be considered consequences of genetic damage because they were not exposed to environmental irradiation from the contaminants. The morphological abnormalities identified in the fifth generation may have resulted from a mixture of physiological and genetic damages.

The second criterion is that the abnormality rates were primarily caused by radioactive cesium when the rates were correlated with the measured ground radiation doses (Fig. 5b). This is because the ground radiation doses we measured were mainly from ^137^Cs and ^134^Cs and not from short-lived radionuclides (such as ^131^I), which would have decayed by the time of our field survey. These cesium species were not distributed in a concentric fashion around the NPP, whereas the high-energy short-lived radionuclides, such as ^131^I, were distributed in a concentric fashion from the NPP [71]. Thus, when the abnormality rate was correlated with the distance from the NPP, the findings indicate that the damage was mainly the result of short-lived radionuclides (Fig. 5b).

Based on these judgment criteria, the small forewing size and abnormalities detected in the first generation after the NPP accident were caused by physiological damage, including somatic cell DNA damage (Fig. 5c). The forewing size was correlated with the ground radiation dose, which indicates that cesium was mainly responsible for this change. In the first and fifth offspring generations, we identified abnormalities, which indicates that they were likely caused by genetic damage. The abnormalities were correlated with distance from the NPP, which suggests that the genetic damage was likely caused by short-lived radionuclides.

We also noted that the abnormality and mortality rates were always higher in the fifth generation (the fall of 2011) compared with the first generation (the spring of 2011) in both the P and F_1_ generations in all localities, with the exception of a single locality [34, 36]. This result suggests that germ cell DNA damage had accumulated over generations toward the fifth generation (the fall of 2011).

The eclosion time (not shown in Fig. 5) was correlated with the distance from the NPP in both the spring (May) and fall (September) of 2011. Thus, eclosion time changes were likely caused by short-lived radionuclides. The early germline and somatic DNA damage caused by high-energy short-lived radionuclides released immediately after the NPP accident likely contributed to these changes. Taken together, we conclude that the first generation was more severely damaged, in terms of both physiological damage in somatic cells and genetic damage in germline cells, than the subsequent generations.

## MATHEMATICAL MODELS

Biological responses to the amount of cesium radioactivity ingested by larvae were examined in two different experiments, first in Hiyama *et al.* (2012) [34], using leaves contaminated at relatively high levels, and second in Nohara *et al.* (2014) [38], using leaves contaminated at relatively low levels. The experiment shown in Hiyama *et al.* (2012) [34] was further examined mathematically in Nohara *et al.* (2014) [35], which indicated that the response follows a power function model. Here, we analyzed these data together and fit them to a single mathematical model from low to high levels of contamination.

Using a standard statistical analysis tool, R version 3.0.2 (R Foundation for Statistical Computing, Vienna, Austria), we derived mathematical models (linear, logistic, log-logistic, Gompertz, Weibull, and power function) and examined the Akaike's information criterion (AIC) associated with these models. When all 11 available data points were included, a Weibull model (Type 2; 3 parameters) exhibited the lowest AIC value (AIC = 84.76) among the 12 models tested (Table 1).

**Fig. 6.**
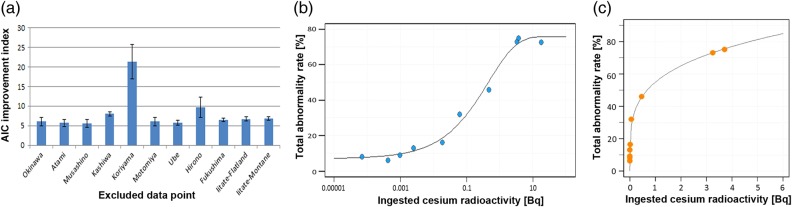
Mathematical analyses of the total abnormality rates (including the deaths of larvae, prepupae, and pupae and the morphological abnormalities of adults), which were obtained from high [34] and low [38] contamination levels, in response to cesium radioactivity ingested by Okinawa larvae. The horizontal axis represents ingested cesium radioactivity per larva [Bq/body]. (**a**) AIC improvement index. The highest AIC improvement index is obtained when the Koriyama data point is excluded as an outlier. (**b**) A Weibull model (type 2, 4 parameters) is the best mathematical fit when the Koriyama data point is excluded. The distribution pattern is loosely sigmoidal. The threshold is approximately 10 mBq/body, and the saturation point is approximately 10 Bq/body. (**c**) A power function model is the best mathematical fit when the Koriyama and Iitate-montane data points are excluded.

We noticed that the Koriyama data point was likely the most deviated from the rest of the points, and this hypothesis was tested by excluding each single point and obtaining the AIC values from all 12 models tested. The five lowest AIC values were selected, and their mean AIC values were obtained. Similarly, for all 11 available data points, the mean AIC values of the five lowest data points were also obtained. By subtracting the mean AIC values of 10 points from those of 11 points, we obtained values that indicate an improvement of the model fit regarding the AIC value (termed AIC improvement index) via the exclusion of a single point. The AIC improvement index varied from 5.61 ± 1.01 (Musashino excluded) to 9.70 ± 2.65 (Hirono excluded), with the exception of one data point (Koriyama excluded) at 21.2 ± 4.39 (Fig. 6a). These results justified the elimination of the Koriyama point as an outlier.

The model fit results after excluding the Koriyama point indicated an overall improvement of the AIC values (Table 2). Among these models, a Weibull model (Type 2; 4 parameters) exhibited the lowest value (AIC = 58.87) (Table 2; Fig. 6b). In general, the Weibull function has been used in survival analyses in many academic disciplines to describe a failure of systems, including mechanical and biological machines. According to this equation, the half abnormality dose (the dose required to cause death or morphological abnormality in 50% of the individuals in the treated group) was calculated at 0.45 Bq/body. There is a loose threshold of ∼10 mBq/body and a saturation point of ∼10 Bq/body (Fig. 6b). From the threshold to the saturation point, three orders of magnitude were required.

**Table 2.** Mathematical models with AIC to fit data points excluding the Koriyama point

Model	Function	AIC
Linear model	f(x)=3.4x+27	95.34
Logistic model (3 parameters)	$f(x)=\frac{74}{1+e^{-4.5(x-0.33)}}$	71.66
Logistic model (4 parameters)	$f(x)=-739+\frac{812}{1+e^{-2.2(x+1.2)}}$	70.39
Log-logistic model (3 parameters)	$f(x)=\frac{92}{1+e^{-0.43(log(x)-log0.29)}}$	65.85
Log-logistic model (4 parameters)	$f(x)=7.6+\frac{73}{1+e^{-0.67(log(x)-log0.26)}}$	62.52
Gompertz model (3 parameters)	$f(x)=73e^{-e^{-3.0(x-0.18)}}$	70.66
Gompertz model (4 parameters)	$f(x)=35-32e^{-e^{1.2(x+65)}}$	104.78
Weibull model (type 1; 3 parameters)	$f(x)=142e^{-e^{-0.17(log(x)-log0.61)}}$	69.40
Weibull model (type 1; 4 parameters)	$f(x)=8.9+78e^{-e^{-0.41(log(x)-log0.13)}}$	64.63
Weibull model (type 2; 3 parameters)	$f(x)=78(1-e^{-e^{0.38(log(x)-log0.40)}})$	62.54
Weibull model (type 2; 4 parameters)	$f(x)=6.8+69(1-e^{-e^{0.52(log(x)-log0.47)}})$	58.87
Power function model	$f(x)=50x^{0.19}$	74.14

The Koriyama data point was removed on the assumption that it was an outlier.

A further point of consideration was that the Iitate-montane point was different from the rest of the points. Because the Iitate-montane point received extremely high levels of cesium, it may be expected that the biological response mechanism of this data point is fundamentally different from that of the other data points, as has been discussed in the previous papers [34–37]. The exclusion of the Iitate-montane point, together with the Koriyama point, resulted in further improvements of the AIC values. Among the models examined, a power function model (AIC = 46.98) was superior to the best Weibull model (AIC = 48.93) (Table 3). This power function equation, *y* = 55*x*^0.24^ (Fig. 6c), is simpler than the Weibull equations, and it is consistent with and similar to our previous results [35]. According to this power function equation, the half abnormality dose was calculated at 0.69 Bq/body, and there was no threshold.

**Table 3.** Mathematical models with AIC to fit data points excluding the Koriyama and Iitate-montane points

Model	Function	AIC
Linear model	f(x)=17x+17	73.30
Logistic model (3 parameters)	$f(x)=\frac{74}{1+e^{-4.5(x-0.34)}}$	66.22
Logistic model (4 parameters)	$f(x)=-1253+\frac{1327}{1+e^{-2.1(x+1.4)}}$	65.22
Log-logistic model (3 parameters)	$f(x)=\frac{173}{1+e^{-0.32(log(x)-log8.9)}}$	50.02
Log-logistic model (4 parameters)	$f(x)=4.9+\frac{127}{1+e^{-0.42(log(x)-log2.4)}}$	49.41
Gompertz model (3 parameters)	$f(x)=74e^{-e^{-3.0(x-0.18)}}$	65.33
Gompertz model (4 parameters)	$f(x)=-741+815e^{-e^{2.1(x+1.2)}}$	65.21
Weibull model (type 1, 3 parameters)	$f(x)=210e^{-e^{-0.41(log(x)-log5.5)}}$	57.11
Weibull model (type 1, 4 parameters)	$f(x)=7.4+203e^{-e^{-0.18(log(x)-log6.5)}}$	48.93
Weibull model (type 2, 3 parameters)	$f(x)=201(1-e^{-e^{0.27(log(x)-log59)}})$	48.68
Weibull model (type 2, 4 parameters)	$f(x)=4.0+102(1-e^{-e^{-0.37(log(x)-log2.4)}})$	49.56
Power function model	$f(x)=55x^{0.24}$	46.98

The Koriyama data point was removed on the assumption that it was an outlier, and the Iitate-montane point was removed on the assumption that the initial phase in low-dose levels and the last phase in high-dose levels follow different models.

These analyses demonstrated that it is possible to coherently understand the biological response (i.e. total abnormality rate) to an ingested cesium dose from low-dose to high-dose ranges according to the Weibull function or power function models.

## QUESTIONS AND ANSWERS

### Q1. Are the results of the pale grass blue butterfly relevant to humans?

The short answer for this question is yes; however, there are also species-specific aspects. Therefore, simple extrapolation to other animals, including humans, is not adequate. If there are important abnormal phenotypes in butterflies, humans would be similarly disturbed at the molecular level. However, we do not know the phenotypic outcomes of humans at the individual level, which would be species-specific.

All organisms share similar molecular mechanisms – referred to as the unity of life. However, all organisms are also unique as species and as individuals within a species. Thus, organisms are highly diverse. Unity and diversity are two key concepts used to understand biological phenomena on the earth, and these concepts are included in most textbooks of the biological sciences [72–78].

Radiation responses are general stress responses that are widely found in organisms. Radiation induces the generation of reactive oxygen species that damage macromolecules, such as DNA and proteins, and this universal biochemical process does not involve a species-specific process. However, butterflies and humans are two different species, and they exhibit different species-specific responses to radiation.

In the history of biochemistry and molecular biology, model organisms, such as *Escherichia coli* (colon bacterium), *Caenorhabditis elegans* (namatode worm) and *Drosophila melanogaster* (fruit fly, an insect), have contributed to our modern understandings of organisms, including humans. In the introduction section of a seminal book in developmental biology published in 1992 [72], Peter A. Lawrence stated this point clearly:

‘It has long been an article of faith amongst biologists that understanding gained by studying one system is likely to apply to others and often this has proved to be the case. When I was a student there was a growing subject in insect biochemistry; it was suspected by some that insects might do things very differently – they might not have a Krebs cycle, for instance. Of course it has turned out that insect and mammalian biochemistry are fundamentally similar.’

(Page xii, Ref. [72]).

Of course, *D. melanogaster* (fruit fly) and *Z. maha* (butterfly) are both insects. In the 21st century, we are confident that many insect-based results at the molecular level are relevant to humans. The biological responses to radiation are basically molecular phenomena. Therefore, at the molecular level, ionization and damaging mechanisms would be similar between butterflies and humans.

### Q2. The experimental conditions for the internal exposure experiments appear to be different from wild conditions. Can these experimental results be extrapolated to the field case of this butterfly?

No, a simple extrapolation is not adequate, because situations in the field may be more severe for the following four reasons. First, larvae consume contaminated leaves in the field throughout their developmental stages, which results in greater internal exposure. In our experiment, the early larvae consume non-contaminated leaves. Second, internal exposure from inhaled air is possibly more severe in the wild. We performed our experiment in a non-contaminated clean environment, although the air inside the containers may have been ‘contaminated’ by radioactive materials released from the surface of the leaves. Third, external exposure is more severe in the wild, although even in our rearing conditions, larvae may have been externally irradiated by the contaminated leaves. Fourth, the wild living environment is more stressful in general [79], which is most likely because of changeable climates and predation pressure.

### Q3. When are germline cells produced in this butterfly? Depending on this information, is it possible that internal exposure may not effectively cause germline DNA damage?

In insects, germline cells are allocated at the earliest stage of embryogenesis [80, 81]. At the later instar stages, germline cells undergo active division, growth and differentiation. It is during this period that the sensitivity to ionizing radiation may be the highest. In the pupal stage, eggs and sperms are almost mature. We have not examined this stage in the pale grass blue butterfly; however, we presume that these general facts in insects are applicable to this butterfly. Thus, theoretically, internal exposure through ingestion could cause germline DNA damage. However, we believe that it is rare to find germline mutations caused by low-level chronic exposure from an ingested diet.

### Q4. In Hiyama ***et al.*** (2012) [34], the control leaves for the internal exposure experiment were collected from Ube, Yamaguchi Prefecture. Because this city is located at a considerable distance from Fukushima, the biochemical character of the Ube leaves may be inherently different from the Fukushima leaves. Ideally, the best control would be the non-contaminated leaves from Fukushima. Do you have such a control?

Yes, we do now. This question states that the host plant leaves in the Kanto-Tohoku districts may be inherently toxic to Okinawa larvae, even without contamination. The host plant *O. corniculata* is globally distributed, and we do not believe that leaves from Ube are significantly different from those from Fukushima. We agree that non-contaminated leaves from Fukushima would be the best control; however, these leaves were not available at the time, and we used the non-contaminated Ube leaves as a control in Hiyama *et al.* (2012) [34]. In 2013, Fukushima leaves exhibited substantially lower contamination levels compared with leaves from previous years. Therefore, Fukushima leaves were used in recent experiments, and differences from Ube and Okinawa leaves were difficult to detect (unpublished data).

Additionally, in another study [38], we used leaves with low contamination levels from Atami and other cities that are substantially closer to the NPP than Ube but reasonably far from the NPP and did not observe detectable effects. We also reared larvae from many different localities in Japan (including Aomori, Akita, Fukushima, Ibaraki, Hyogo, Yamaguchi and Kagoshima) with Okinawa leaves, and we did not detect a significant effect. Therefore, we are confident that leaves from Ube or anywhere in Japan are essentially biochemically identical to the Fukushima leaves for this butterfly species, with the exception of the Fukushima leaves that were contaminated by the radioactive materials from the NPP.

### Q5. How did you define ‘abnormality’? Are your judgment criteria arbitrary or subjective?

No, our judgment criteria are not arbitrary or subjective. Abnormal patterns are defined as patterns that deviated from the basic color pattern of this species of butterfly as defined in Iwata *et al.* (2013) [31]. We have examined this butterfly for more than a decade, and we are confident that we have accumulated sufficient knowledge to provide objective judgments. We counted only morphological abnormalities that were obviously different from the normal counterparts, and ambiguous abnormalities were excluded. The morphology was examined by two examiners in the first paper [34]. We believe that our judgments of abnormality and their associated values are completely accurate or underestimated, because clear cases of morphological abnormalities may have been overlooked, as noted by one of the examiners. We did not perform blind tests because it is not necessary for these clear cases. Moreover, counting the number of dead individuals cannot be biased. Death is the ultimate physiological result for organisms, and it is the most indicative output for us.

### Q6. Were the abnormal wing color patterns found in individuals collected in Fukushima confused with temperature-shock (TS) type modifications?

No, the patterns were not confused, because the Fukushima abnormality and TS-type modifications are very different. Detailed discussions can be found in other papers [36, 37]. Additionally, see Q12 for comparisons with the EMS-induced changes. In scoring abnormal individuals with respect to the wing color patterns, we excluded individuals with color patterns equivalent to the TS types.

In reality, we identified only ∼20 adult individuals with weak TS-type modifications (one exception was a strong outward TS type from Hirono caught in the spring of 2013) in 2191 field-collected adults over 3 years (six sets of field surveys). Thus, the percentage of TS-type adults obtained in the Kanto-Tohoku districts was 0.9%. We previously estimated that the frequency of TS types in Kamakura, Kanagawa Prefecture, was of the order of 0.1% [32]. Thus, the value of 0.9% was slightly higher than the estimated percentage, but within the order of the previous estimate. We do not know if this difference is significant; however, if it is significant, the difference would be related to the temperatures in the Kanto-Tohoku districts surveyed, which were colder than the temperatures in Kamakura.

In the offspring generations generated in our laboratory, <30 adult individuals with weak TS-type modifications were obtained out of 9510 reared individuals over 2.5 years (five sets of laboratory rearing experiments). Thus, the percentage of TS-type adults obtained under the 25°C laboratory conditions was 0.3%, which is lower than the field value of 0.9%. This finding indicates that field conditions with temperature fluctuations may have some influence on the emergence of the TS types. In addition, the results were not zero, even under the 25°C laboratory conditions, which suggests the involvement of genetic components in the occurrence of the TS-type modifications.

### Q7. Are there any reasons why correlations were not found between ground radiation dose and abnormality rate in the spring of 2011?

The fact that correlations were not identified between the ground radiation dose and the abnormality rate in the spring of 2011 most likely indicates that these abnormalities were not mainly caused by cesium species. The abnormalities detected in the spring of 2011 were likely introduced by both short-lived radionuclides and cesium together, which makes it difficult to obtain good correlations.

### Q8. How do you explain the accumulation of damage toward the fifth generation?

The accumulation of harmful effects in 2011 and 2012 may have been caused by genetic damage resulting from the initial exposure immediately after the NPP accident from high-energy short-lived radionuclides. We believe that additive chronic exposure also contributed to the gradual accumulation of germline damage. However, the damage occurring after the fall of 2011 might not have caused serious genetic damage in the population.

A further explanation for the delayed peak is genomic instability (which has a detrimental effect on genomes in non-irradiated subsequent generations [56–59]) caused by short-lived radionuclides immediately after the NPP accident.

### Q9. Was the real peak of abnormality and mortality located earlier, before the fall of 2011?

We examined the adult morphology of the 1st, 5th, 7th, 11th, 13th and 17th generations after the NPP accident in seven localities, and data are not available between these generations. In our study, a peak was identified at the 5th generation in most localities. However, a peak was identified at the 7th generation in certain localities. The peak time appears to have been influenced by the locality or the effect on a given locality. Thus, it is likely that the integrated peak of the examined localities may have been close to the 5th generation.

We evaluated the mortality rates of the offspring from parents caught in the field. A very high mortality was identified in the 5th generation (the fall of 2011) in many localities. In Hirono, the mortality rate was >90%. Peaks higher than this rate would entirely eradicate the population. Based on these results, we believe that the actual peaks of many contaminated localities were located in the generations close to the 5th generation.

### Q10. Do you think that different peak times between the ground radiation dose and the biological responses (i.e. abnormality and mortality rates) can be explained by the low-level internal exposure of the first generation as a result of the small amount of leaves ingested after the overwintering period?

No, we do not agree with this speculation because it is highly likely that the overwintering larvae consume more leaves overall than the non-overwintering larvae. The overwintering larvae appear to become the fifth instar in the spring and consume a large amount of leaves. In contrast, non-overwintering larvae become pupae after the fourth instar.

Moreover, we believe that the high-energy short-lived radionuclides that occurred only immediately after the accident had a much greater contribution to the abnormality and mortality rates than the ingestion of radionuclides at later times. The impact on the first generation after the NPP accident was most likely substantially larger than the impact on the second and subsequent generations.

However, the developmental stages of overwintering larvae may vary, which indicates that the consumption of contaminated leaves may vary among the overwintering larvae. This variation might have increased the difficulty of obtaining a good correlation between the ground radiation dose and the abnormality rate in the first generation. Furthermore, many first-generation individuals may have died before or immediately after eclosion, and this may have also increased the difficulty of collecting abnormal individuals from the field.

### Q11. Are the abnormalities found in the internal exposure experiments likely to have resulted from defects caused by physiological stress?

Yes. Our internal exposure experiments are designed to examine physiological (somatic) effects and not to examine heritability *per se*. Accordingly, in these experiments, we basically examined only the generation of Okinawa larvae that directly consumed the contaminated leaves (with the exception of the successive feeding experiment). We have not performed experiments to examine the heritability of abnormalities induced by the ingestion of contaminated leaves. Thus, the abnormalities identified in the internal exposure experiments represent physiological damage, which most likely includes somatic mutations, and not germline damage.

In addition, the successive feeding experiment, in which healthy F_1_ adults were selected to generate the offspring (F_2_) generation, was conducted to examine physiological damage. Regardless of what the F_1_ generation consumed, a high mortality in the F_2_ generation was detected when contaminated leaves were fed [38]. However, when non-contaminated leaves were fed, the F_2_ generation exhibited low (normal) levels of mortality [38]. Thus, the larval diets rather than their genetic makeup are highly influential, which suggests that the high mortality in this series of experiments was not caused by germline DNA damage, even in the F_2_ generation. In a sense, these results may not be surprising because healthy adults were selected as parents for the offspring generation; however, it is still important to estimate the physiological effects in the offspring generation (born from healthy parents) who consumed contaminated leaves.

However, the forewing size, which is representative of physiological stress from irradiation in the F_1_ generation, was transgenerational. This result suggests that even if there is no germline damage, stress effects can indirectly alter the robustness of the next generation, such as through malnutrition and epigenetic molecular changes in the germline cells.

### Q12. How are the abnormalities detected in the field or in reared samples similar to the EMS-induced abnormalities?

In the F_1_ generation from the EMS-treated adults, we identified the following eight phenotypes that were likely to have resulted from germline damage: dorsoventral transformation (DV), background gap (BG), weak contrast (WC), disarrangement of spots (DS), reduction of spots (RS), loss of spots (LS), fusion of spots (FS), and ectopic spots (ES) [31]. We demonstrated that these aberrant phenotypes were inherited by the F_2_ generation [31].

We have identified the following aberrant phenotypes in the Fukushima samples: WC, RS, LS, ES, DS, FS, deformation of spots (DfS), misplacement of spots (MS), and enlargement of spots (EnS). These aberrant phenotypes are collectively similar to the EMS-treated F_1_ phenotypes. The DfS, MS and EnS are unique to the Fukushima samples. These data strongly suggest that the Fukushima's wing color pattern aberrations are produced via germline DNA damage similar to that of the EMS treatment. However, the DV and BG phenotypes do not occur in the Fukushima samples. Because these phenotypes are relatively severe, the Fukushima damage may be less severe than the EMS-induced damage.

Another related point is that the EMS-induced aberrant phenotypes and the Fukushima individuals exhibited no rule of changes, which is different from the TS-type modifications. For example, only a single spot is changed or a small area is affected in the Fukushima and EMS-treated samples, whereas in the TS types, predictable changes occur that are coordinated throughout a single wing or throughout four wings in an individual.

### Q13. How do you explain the sensitivity difference between the F_1_ and F_2_ generations found in Nohara ***et al.*** (2014) [38]? (Here, ‘sensitivity difference’ means the difference in abnormality rate or mortality rate in response to the ingestion of contaminated leaves at the various levels.)

Laboratory-reared individuals generally become weak after a number of generations; thus, it was not surprising to find that the F_2_ generation was more sensitive than the F_1_ generation. Although the F_1_ effect is not entirely null, the increase in sensitivity may have been caused by conditional differences between the laboratory and the field. The lack of environmental stress (e.g. constant ‘climate’ conditions) and natural selection in a laboratory may increase the vulnerability of laboratory individuals to stress.

### Q14. Are the physiological effects (somatic effects) of ingesting contaminated leaves greater than the genetic effects?

In the experimental system presented in Nohara *et al.* (2014) [38], the physiological effects were greater; however, such results are dependent on the experimental systems. In our system, we selected healthy individuals to produce offspring to test the physiological impacts from ingestion, and individuals with greater genetic damage may have died before the adult stage. Thus, our results cannot exclude genetic impacts.

### Q15. Do you think that ingestion below the threshold you observed at 10 mBq/body in the dose–response curve of the Weibull function model affects the organisms?

We believe that the biological responses below and above the threshold are not particularly distinct and should be viewed with caution. First, this threshold can be identified only after logarithmic transformation. Second, at the non-transformed scale, the total abnormalities slightly increased, even below this threshold, from Ube to Atami and Musashino. Third, the entire dataset can also be fitted to a power function model, which eliminates the relevance of the thresholds. Fourth, strain dependence or individual dependence may have impacted the sensitivity to radioactivity. Fifth, even if this ‘threshold’ occurs, the 10-mBq/body boundary could easily shift to 1 mBq/body if additional data points are accumulated. Sixth, the output is the ‘total abnormality rate’, to which deaths significantly contributed. Thus, smaller impacts would be expected, even below the ‘threshold’ level.

### Q16. Is the normality attained after the peak of mortality and abnormality because of adaptive evolution or a decline in environmental radiation levels?

Adaptive evolution here is defined as changes in the frequency of genes that confer radiation resistance to butterflies at the population level. We believe that the normality attained after the peak of mortality and abnormality was mainly caused by random exposure at the individual level and possibly because of adaptive evolution. The level of exposure of an individual depends on chance and varies considerably within a population. ‘Unlucky’ individuals that were heavily exposed to radiation would be eliminated from the population, leading to a negative selection. However, among the individuals that received considerable exposure, some were stronger than others because their genetic profile conferred radiation resistance to the individuals. Furthermore, because radiation is a mutagen that may produce new alleles for radiation resistance, exposure may lead to a positive selection for these alleles.

Of course, we think that the decline in radioactive materials may also have contributed to the recovery of normality. However, the decline in radioactive materials cannot be the sole explanation for the normality attained following the disastrous damage observed at the population level, because of the following three points. First, even in the fall of 2013, the residual radioactivity was significant, and the radioactive materials were not entirely eliminated. For example, the residual activity in Fukushima in the fall of 2013 was as high as the activity in the spring of 2011 according to our survey [39]. And the residual activity in Fukushima in the fall of 2013 was much higher than the activity in the spring of 2011 in Iwaki, where the impact was clearly observed. Second, the dynamics of abnormality are very different from the dynamics of radioactivity. Radioactivity levels decreased after the accident, whereas the abnormality rate increased gradually toward the fall of 2011 or the spring of 2012 and subsequently decreased. Third, the radiation level of Motomiya in the fall of 2013, for example, was substantially higher than that in Tsukuba in the spring of 2011. Thus, Tsukuba's level in the spring of 2011 would be too low to induce detectable abnormality and mortality if the Motomiya result in the fall of 2013 was entirely caused by a decline in radioactive levels. However, Tsukuba's butterflies responded to the low levels, and in the fall of 2013, Motomiya's butterflies no longer responded to this level of radiation. Although certain evidence is consistent with adaptive evolution, we admit that further research is required to clearly demonstrate the adaptive evolution of the radiation-exposed populations of the pale grass blue butterfly.

### Q17. Are there any known cases of adaptive evolution similar to this case?

Yes, prior cases of adaptation to environmental radiation have been established [82, 83]. More generally, the story of the peppered moth is likely the most famous case of adaptive evolution. In this case, industrial chemicals, not radioactive materials, initiated adaptive evolution [84–87]. However, industrial chemicals are not mutagenic (and thus do not induce genetic changes), and that selection process was dependent on predators. In the case of the pale grass blue butterfly in Fukushima, radiation acted as a mutagen (that induced genetic changes) and simultaneously functioned as a selective pressure and an environmental stressor. We believe that many butterflies have evolved in a similar way in response to environmental stress [88–90]. However, the adaptive evolution of the pale grass blue butterfly has not been experimentally demonstrated. See Q16 for an additional discussion.

## CONCLUSIONS

The following important points were reviewed regarding the ingestional impacts and transgenerational effects of the Fukushima nuclear accident. (i) The consumption of contaminated foods, even at low doses, is harmful to many individuals of the pale grass blue butterfly. The ingestional impacts may originate from radiation stress as well as physical stress from metallic microparticles. (ii) Damage introduced by the ingestion of contaminated foods is largely physiological; however, it may also be transgenerational through epigenetic changes. (iii) Radiation sensitivity varies within this species and is the basis for adaptive evolution to radiation resistance. (iv) Biological effects (i.e. deaths and abnormalities) in response to an ingested cesium dose are best fit to the Weibull function or power function models. In the former, a loose threshold occurs at ∼10 mBq/body, and the saturation point is ∼10 Bq/body. (v) Harmful effects in the offspring generation may be enhanced by the ingestion of contaminated foods, but they may be suppressed by the ingestion of non-contaminated foods. (vi) Germline DNA damage was introduced immediately after the explosions at the NPP, and it was inherited by subsequent generations. This germline DNA damage was most likely introduced by high-energy short-lived radionuclides during the late larval stage at the level of the ground surface. (vii) Cesium is likely primarily responsible for the physiological damage. (vii) Damage, including germline mutations, accumulated until the fall of 2011. (ix) Individuals with severe radiation-induced damage have been eliminated, and the population regained normality after ∼15 generations within 3 years.

## FUNDING

This study was funded by the Takahashi Industrial and Economic Research Foundation, the Sumitomo Foundation, the Asahi Glass Foundation, act beyond trust, the University of the Ryukyus and generous private donors who care about the wildlife and individuals who live in the contaminated areas affected by the Fukushima NPP accident. Funding to pay the Open Access publication charges for this special issue was provided by the Grant-in-Aid from the Japan Society for the Promotion of Science (JSPS) [KAKENHI Grant No. 26253022].
